# Integrins control epithelial stem cell proliferation in the *Drosophila* ovary by modulating the Notch pathway

**DOI:** 10.3389/fcell.2023.1114458

**Published:** 2023-02-28

**Authors:** Lourdes Rincón-Ortega, Andrea Valencia-Expósito, Anna Kabanova, Acaimo González-Reyes, Maria D. Martin-Bermudo

**Affiliations:** Centro Andaluz de Biología del Desarrollo, CSIC/Universidad Pablo de Olavide/JA, Sevilla, Spain

**Keywords:** integrin, proliferation, Notch, laminin, stem cell

## Abstract

Cell proliferation and differentiation show a remarkable inverse relationship. The temporal coupling between cell cycle withdrawal and differentiation of stem cells (SCs) is crucial for epithelial tissue growth, homeostasis and regeneration. Proliferation vs. differentiation SC decisions are often controlled by the surrounding microenvironment, of which the basement membrane (BM; a specialized form of extracellular matrix surrounding cells and tissues), is one of its main constituents. Years of research have shown that integrin-mediated SC-BM interactions regulate many aspects of SC biology, including the proliferation-to-differentiation switch. However, these studies have also demonstrated that the SC responses to interactions with the BM are extremely diverse and depend on the cell type and state and on the repertoire of BM components and integrins involved. Here, we show that eliminating integrins from the follicle stem cells (FSCs) of the *Drosophila* ovary and their undifferentiated progeny increases their proliferation capacity. This results in an excess of various differentiated follicle cell types, demonstrating that cell fate determination can occur in the absence of integrins. Because these phenotypes are similar to those found in ovaries with decreased laminin levels, our results point to a role for the integrin-mediated cell-BM interactions in the control of epithelial cell division and subsequent differentiation. Finally, we show that integrins regulate proliferation by restraining the activity of the Notch/Delta pathway during early oogenesis. Our work increases our knowledge of the effects of cell-BM interactions in different SC types and should help improve our understanding of the biology of SCs and exploit their therapeutic potential.

## 1 Introduction

The processes of stem cell proliferation and differentiation are intimately entwined, as the latter is usually accompanied by irreversible cell cycle exit. The temporal coupling between cell cycle withdrawal and differentiation of stem cells is crucial for normal epithelial tissue growth and development, and continues to be critical for tissue homeostasis and regeneration throughout life. A failure to arrest stem cell proliferation and to enter into differentiation can lead to abnormal tissue development, a variety of diseases and is a hallmark of cancer cells.

Accumulated knowledge over the last decade has demonstrated that the extracellular matrix (ECM) and its main receptors, the integrins, are key regulators of stem cell proliferation and differentiation during development. However, the picture is highly complex and far from understood, as there is considerable variation in the type of integrins and interacting ECM components in the different tissues. Thus, while integrin-ECM interactions maintain the self-renewal capacity of various somatic stem cell types, including epidermal (Zhu et al., 1999), neural (Campos et al., 2004) and hematopoietic (Williams et al., 1991), they promote the differentiation of mouse embryonic stem cells (Hayashi et al., 2007). These opposing activities are likely to be mediated by the ability of integrins to activate unique, stem cell type-dependent intracellular signaling pathways. However, the mechanisms involved are incompletely comprehended.

**FIGURE 1:**
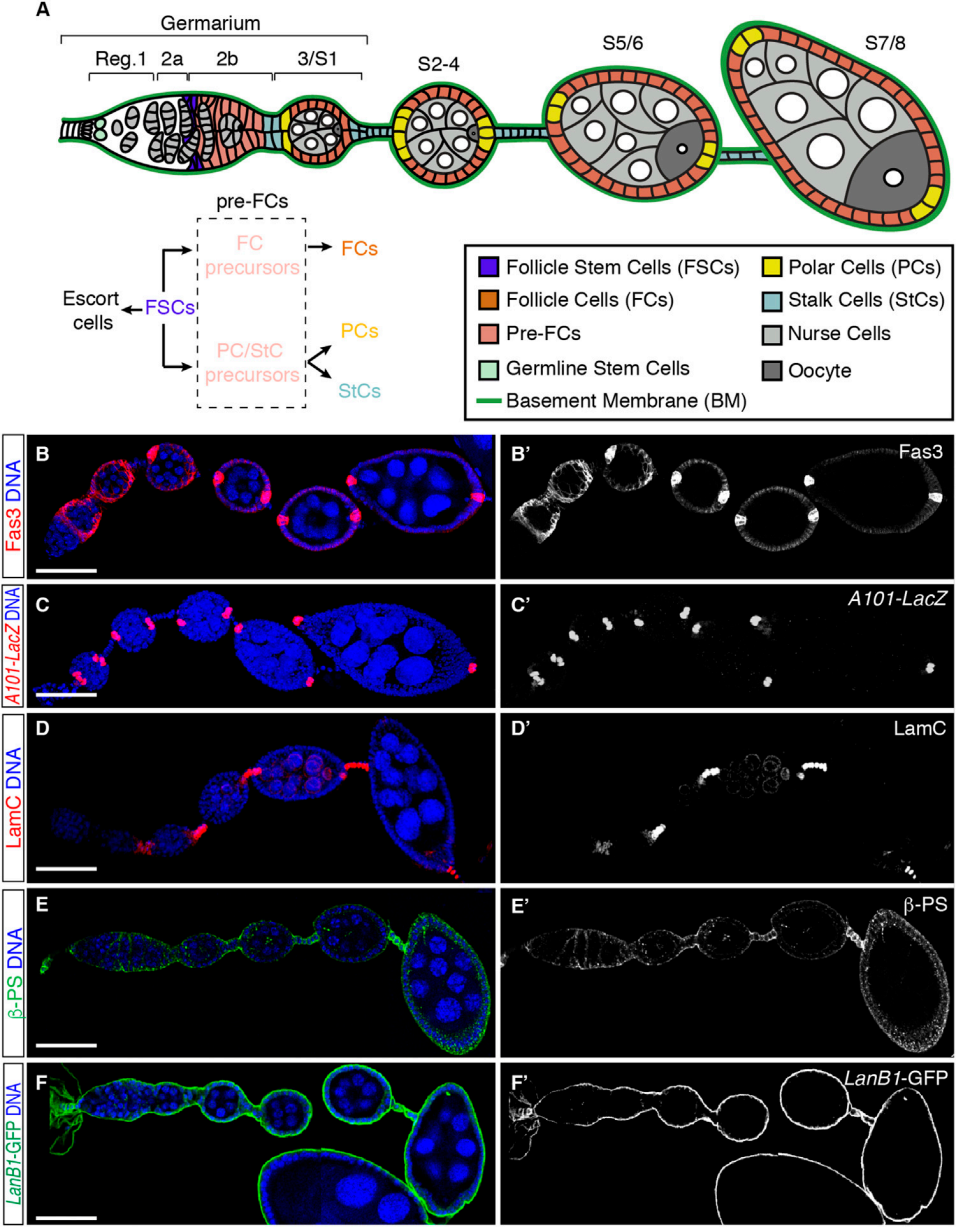
Cellular organisation of the initial stages of oogenesis. **(A)** Scheme of the anterior half of an ovariole showing regions 1, 2a, 2b, and 3 of the germarium and developing egg chambers up to stage (S) 7/8. Different cell types and the basement membrane (BM) are indicated. Follicle stem cells (FSCs) proliferate and give rise to escort cells and pre-follicle cells, the latter composed of follicle cell (FC) and polar cell (PC)/stalk cell (StC) precursors, ultimately responsible for the generation of FCs, PCs and StCs. Germline cysts are composed of 15-nurse cells and 1 oocyte. **(B–F)** ovarioles stained to show the pattern of expression of Fas3 [somatic cells from region 2a onwards, accumulated in PCs from S3/4 onwards; **(B,B**′**)**], *A101-LacZ* [labels PCs; **(C,C**′**)**], LamC [marks StCs; **(D,D**′**)**] and ßPS [Myospheroid; **(E,E**′**)**]. LanB1-GFP labels the BM **(F,F**′**)**. Scale bars: 50 µm.

The follicular epithelium (FE) of the *Drosophila* ovary constitutes an excellent model system to study the role of cell-ECM interactions in stem cell proliferation and differentiation during development. The *Drosophila* ovary is composed of about 15 ovarioles, each containing a germarium at their anterior end and progressively older egg chambers towards their posterior end, all surrounded by a basal BM. The germarium is divided into 3 regions. Region 1 is the mitotically active area where the germline cysts are formed upon 4 synchronous mitoses of the cystoblast. Region 2 includes the 16-cell cysts and is subdivided into 2 regions, 2a and 2b, the latter characterized by the lens-like shape of the 16-cell cysts—which span the entire width of the germarium—and their transition to the spherical cysts characteristic of region 3, also known as stage 1 (S1) of oogenesis (Figure 1A). Oogenesis takes roughly a week and has been arbitrarily divided into 14 stages based on morphological criteria, from S1 when egg chambers bud from the germarium to S14, the mature egg [Figure 1A, reviewed in (Bastock and St Johnston, 2008)]. Each egg chamber is composed of 16-cell germline cysts containing 15 nurse cells (NCs) and one oocyte (Oo) surrounded by a single layer of follicle cells (FCs), which constitute the follicular epithelium (FE). FCs originate from a population of stem cells, the Follicle Stem Cells (FCSs), which lie at the border between regions 2a and 2b of the germarium and express low levels of Fas3 [Figures 1A, B; (Margolis and Spradling, 1995; Zhang and Kalderon, 2001; Reilein et al., 2017)]. The FSC population proliferate and diversify to produce escort cells and pre-follicle cells, the latter also expressing Fas3 but at higher levels than FSCs. Pre-follicle cells comprise two distinct cell lineages: 1) the epithelial FC precursors, which proliferate until S6 and generate most of the cells that surround each cyst, and 2) the polar/stalk precursors, which exit mitosis at S1 to S2 of oogenesis and give rise to the polar cell clusters [PCs; visualized with the *A101-LacZ* transgene (Ruohola et al., 1991)] (Figure 1C), located at the anterior and posterior poles of the egg chamber, and to stalk cells [StCs; visualized with an anti-LamC antibody (Pearson et al., 2016)] (Figure 1D) that separate each cyst from the adjacent one (Margolis and Spradling, 1995; Tworoger et al., 1999). These different cell types arise asynchronously and involve activation of the Notch/Delta and JAK/Stat pathways (Torres et al., 2003). As a new cyst buds from the germarium in region 2b, Delta signal from the germline activates Notch in the adjacent anterior polar/stalk precursors, inducing them to develop as anterior PCs (aPCs). aPCs turn on the JAK/Stat ligand Unpaired and induce more anterior polar/stalk precursors to differentiate as StCs (Silver and Montell, 2001; Beccari et al., 2002; Xi et al., 2003). StCs intercalate with each other to generate a two cell-wide stalk. Posterior PCs (pPCs) differentiate at S2, 24 h after the interfollicular stalks have formed. PCs are produced in excess and their numbers are refined by apoptosis to exactly 2 by S5 (Besse and Pret, 2003; Borensztejn et al., 2018). PCs act as an organizer of terminal FC patterning. Thus, while pPCs confer posterior identity, aPCs specify each of the three distinct anterior terminal fates, the cluster of 6–8 border cells, a population of about 40–50 stretched cells and 30–40 centripetal cells (Grammont and Irvine, 2002). StC numbers are also produced in excess and restricted by limited apoptosis during oogenesis (Borensztejn et al., 2018).

Although there are at least 8 integrin *β* subunits and 18 *α* subunits in vertebrates, the *Drosophila* genome contains only 2 *β* subunits, *β* PS and βν, and 5 *α* subunits, αPS1 to αPS5 (Yee and Hynes, 1993; Brown, 2000). Encoded by the *myospheroid* (*mys*) gene, βPS is the only *β* chain present in the ovary. This subunit is expressed in the germarium, in the germline until stage 3–4, in the follicular epithelium, and at higher levels in the interfollicular stalks [Figure 1E, (Fernández-Miñán et al., 2007)]. βPS integrins regulate several cellular processes in differentiated FCs, including spindle orientation (Fernández-Miñán et al., 2007), establishment and maintenance of cell polarity and shape (Fernandez-Minan et al., 2008; Santa-Cruz Mateos et al., 2020), cell cycle exit to differentiation switch (Gomez-Lamarca et al., 2014) and collective cell migration (Diaz de la Loza et al., 2017). In addition, integrins play independent roles in controlling FSC anchoring and proliferation rates, mainly through interactions of the αPS1 βPS with its primary ligand, laminin, which is present in the basement membrane that surrounds each ovariole [Figure 1F; (Diaz de la Loza et al., 2017)]. Thus, FSC lacking either βPS, αPS1 or *lanA* (which codes for one of the *Drosophila* Laminin A chains) divide less frequently than wild type FSCs. Furthermore, loss of integrins in FSCs causes defects in their progeny, the pre-follicle cells, which include aberrant cell shape and absence of basal domain (O´Reilly et al., 2008). Integrin-LanA interactions are not required to maintain other ovarian stem cell populations, such as germline stem cells, suggesting that unique pathways might regulate niche-stem cell communication in the same organ (O´Reilly et al., 2008). Finally, expression of an RNAi against *mys* in the germarium, in both pre-follicle cells and escort cells, resulted in gross disorganisation of ovarioles, with multilayering in the FE, malformation of interfollicular stalks and incomplete encapsulation and fusion of germline cysts (Lovegrove et al., 2019).

In this work, we have revisited the role of integrin-laminin interactions in the establishment of the different cell types that comprise the FE. In contrast to previous results (O´Reilly et al., 2008), we show that mosaic egg chambers carrying integrin mutant FCs or reduced laminin levels exhibit increased number of polar and stalk cells compared to controls. This phenotype is detected as early as S2. Our results also indicate that integrin function is required in the germarium to restrict proliferation of PC and StC precursors. In addition, we find that integrin-mediated somatic cell-BM interactions controls the size of border cell clusters. Finally, our results suggest that integrins regulate the activation of the Notch pathway to achieve proper PC and StC determination. Hence, our work reveals that cell-BM interactions mediated by integrins contribute to restrict the number of pre-follicle cell precursors, which in turn allows the correct patterning of the FE.

## 2 Materials and methods

### 2.1 Fly stocks

The following fly stocks were used: *mys* ^*11*^ [also known as *mys* ^*XG43*^; (Bunch and Brower, 1992)], *e22c-Gal4 UAS-flipase* (Duffy et al., 1998), *traffic-jam-Gal4* [*tj-Gal4*; (Abdelilah-Seyfried et al., 2003)], *UAS-LanB1 RNAi* (VDRC Cat# 23119), *LanB1::GFP* [Vienna *Drosophila* Resource Centre Cat# 318180; (Sarov et al., 2016)], *neur* ^*A101*^ *-LacZ* (Bloomington *Drosophila* Stock Centre (BDSC) Cat# 4369; (Bellen et al., 1989), *E*(*spl*)*m7-LacZ* (a gift from Prof. Sarah Bray, University of Cambridge, United Kingdom) and *Dl* ^*7*^ (BDSC Cat# 485). The *e22c-Gal4* driver is expressed in somatic cells of the germarium in the pupal and adult ovaries. It was used in combination with *UAS-flipase* and with *y w Ubi-GFP FRT-101* (BDSC Cat# 5153) and *mys* ^*11*^ *FRT-101* to generate *mys* clones, or with *y w v FRT-101* (BDSC Cat# 1844) to generate GFP clones, prior to eclosion and during adult oogenesis. *tj-Gal4* is expressed in most of the somatic cells of the ovarioles and was used to reduce Laminin levels in the basement membrane surrounding the ovarioles (Diaz de la Loza et al., 2017).

### 2.2 Immunohistochemistry

2–4-day old *Drosophila* females grown at 25°C were yeasted for 2 days before dissection. Adult ovaries were dissected at room temperature (RT) in Schneider’s medium (Sigma Aldrich), fixed in 4% paraformaldehyde in PBS (ChemCruz) for 20 min, permeabilized 30 min in PBT and blocked 1 h in PBT-10. Incubation with primary antibodies was performed overnight at 4°C in PBT-1. The following primary antibodies were used: mouse anti-Fas3 (1:50, Developmental Studies Hybridoma Bank (DSHB) Cat# 7G10), mouse anti-βPS (1:50, DSHB Cat# CF-6G11), mouse anti-LamC (1:30, DSHB Cat# ADL84.12) and mouse anti-NICD (1:100, DSHB Cat# C17.9C6), mouse anti-βGal (1:1000, Promega Cat# Z378A), chicken anti-GFP (1:600, Abcam Cat# 13970), rabbit anti-phospho-Histone H3 (1:250, Sigma Aldrich Cat# 06–570) and rabbit anti-Dcp1 (1:100, Cell Signaling Technology Cat# 9578). Secondary antibodies were incubated for 2 h in PBT-0.1 and used 1:200: anti-chicken Alexa Fluor 488 (Invitrogen Cat# A11039), anti-mouse cy3 (Jackson ImmunoResearch Cat# 115-165-146) and anti-rabbit cy5 (Life Technologies Cat# A11008). To label DNA, ovaries were incubated for 10 min with Hoechst (Sigma-Aldrich, 5 mg/ml; 1:1000 in PBT). Ovaries were mounted in Vectashield (Vector Laboratories).

PBT-10: PBS, 10% BSA, 0.1% tween20.

PBT-1: PBS, 1% BSA, 0.1% tween20.

PBT-0.1: PBS, 0.1% BSA, 0.1% tween20.

PBT: PBS (phosphate-buffered saline), 0.1% tween20.

### 2.3 Imaging, processing and quantification of samples

Images were acquired with Leica’s SPE, TCS-SP5 or Stellaris confocal microscopes, analysed utilising ImageJ, and processed with Adobe Photoshop and Adobe Illustrator. Z stacks of fixed samples were taken at 1 μm intervals using 40x/1.3 and 63x/1.4 NA oil immersion objectives.

### 2.4 Statistical analysis

Experimental data correspond to at least three biological replicates. Samples were collected from at least 5 different females grown in equivalent environmental conditions. The arithmetic mean and the standard deviation (SD) of the different experimental settings are shown in the dot plots. Sample sizes correspond to the number of egg chambers or germaria analysed. Statistically significant differences between control and experimental samples were calculated with the non-parametric Mann-Whitney U test. * = *p*< 0.05; ** = *p*< 0.005; *** = *p*< 0.0005; **** = *p*< 0.0001.

### 2.5 Experimental genotypes

#### 2.5.1 Figure 1

(B, D, E) *y w.*

(C) *neurA101-LacZ*.

(F) *LanB1:GFP.*

#### 2.5.2 Figure 2

(A) *y w Ubi-GFP FRT-101*/FMZ; *e22c-Gal4 UAS-flp*/+; *neur* ^*A101*^ *-LacZ/+*

(B) *y w Ubi-GFP FRT-101*/*mys* ^*11*^ *FRT-101*; *e22c-Gal4 UAS-flp*/+; *neur* ^*A101*^ *-LacZ/+*

(D) *y w Ubi-GFP FRT-101*/FMZ; *e22c-Gal4 UAS-flp*/+

(E) *y w Ubi-GFP FRT-101*/*mys* ^*11*^ *FRT-101*; *e22c-Gal4 UAS-flp*/+

#### 2.5.3 Figure 3

(A, E) *y w Ubi-GFP FRT-101*/FMZ; *e22c-Gal4 UAS-flp*/+; *neur* ^*A101*^ *-LacZ/+*

(B, F) *y w Ubi-GFP FRT-101*/*mys* ^*11*^ *FRT-101*; *e22c-Gal4 UAS-flp*/+; *neur* ^*A101*^ *-LacZ/+*

(C) *y w Ubi-GFP FRT-101*/FMZ; *e22c-Gal4 UAS-flp*/+

(D) *y w Ubi-GFP FRT-101*/*mys* ^*11*^ *FRT-101*; *e22c-Gal4 UAS-flp*/+

#### 2.5.4 Figure 4

(C, D) *y w Ubi-GFP FRT-101*/*y w v FRT-101*; *e22c-Gal4 UAS-flp*/+

(E) *y w Ubi-GFP FRT-101*/*mys* ^*11*^ *FRT-101*; *e22c-Gal4 UAS-flp*/+

#### 2.5.5 Figure 5

(B) *y w Ubi-GFP FRT-101*/FMZ; *e22c-Gal4 UAS-flp*/+; *E*(*spl*)*m7-LacZ/+*

(C) *y w Ubi-GFP FRT-101*/*mys* ^*11*^ *FRT-101*; *e22c-Gal4 UAS-flp*/+; *E*(*spl*)*m7-LacZ/+*

(E) *y w Ubi-GFP FRT-101*/*mys* ^*11*^ *FRT-101*; *e22c-Gal4 UAS-flp*/+

#### 2.5.6 Figure 6

(A, F) *y w Ubi-GFP FRT-101*/FMZ; *e22c-Gal4 UAS-flp*/+; *+/TM6B*.

(B, G) *y w Ubi-GFP FRT-101*/FMZ; *e22c-Gal4 UAS-flp*/+; *Dl* ^*7*^ */+*

(C, H) *y w Ubi-GFP FRT-101*/*mys* ^*11*^ *FRT-101*; *e22c-Gal4 UAS-flp*/+; *+/TM6B*.

(D, I) *y w Ubi-GFP FRT-101*/*mys* ^*11*^ *FRT-101*; *e22c-Gal4 UAS-flp*/+; *Dl* ^*7*^ */+*

#### 2.5.7 Figure S1

(A) *y w Ubi-GFP FRT-101*/*mys* ^*11*^ *FRT-101*; *e22c-Gal4 UAS-flp*/+

#### 2.5.8 Figure S2

(A) *y w Ubi-GFP FRT-101*/FMZ; *e22c-Gal4 UAS-flp*/+

(B) *y w Ubi-GFP FRT-101*/*mys* ^*11*^ *FRT-101*; *e22c-Gal4 UAS-flp*/+

(C) *tj-Gal4*/CyO; *UAS-LanB1 RNAi/+*

#### 2.5.9 Figure S3

(A) *tj-Gal4*/CyO; *neur* ^*A101*^ *-LacZ/+*

(B) *tj-Gal4*/CyO; *neur* ^*A101*^ *-LacZ/UAS-LanB1 RNAi*.

(D) *tj-Gal4*/CyO.

(E) *tj-Gal4*/CyO; *UAS-LanB1 RNAi/+*

#### 2.5.10 Figure S4

(A) *tj-Gal4*/CyO.

(B) *tj-Gal4*/CyO; *UAS-LanB1 RNAi/+*

## 3 Results

### 3.1 Integrin activity is required for the correct determination of polar and stalk cell numbers

In order to define the importance of integrin activity in the establishment of the different cell types that conform the follicular epithelium, we made use of a null allele of the *mys* gene (*mys* ^*11*^) to generate complete loss-of-function *mys* ^*-*^ somatic cells (Bunch and Brower, 1992; Fernández-Miñán et al., 2007). We generated epithelia and interfollicular stalks containing control and *mys* ^*-*^ cells (hereafter referred to as mosaic) utilising the *e22c-Gal4* driver, which allowed the generation of mitotic clones during niche formation in pupal stages and throughout adulthood [Supplementary Figure S1; (Reilein et al., 2021)]. We scored the number of anterior and posterior PCs (aPCs and pPCs, respectively) and StCs present in egg chambers at different stages of development. For ease of interpretation, we grouped the different stages into S2, S3/4, S5/6 and S7/8. Both control and experimental samples carried the *A101-lacZ* transgene to label PCs. To identify StCs, they were stained with an anti-LamC antibody.

**FIGURE 2:**
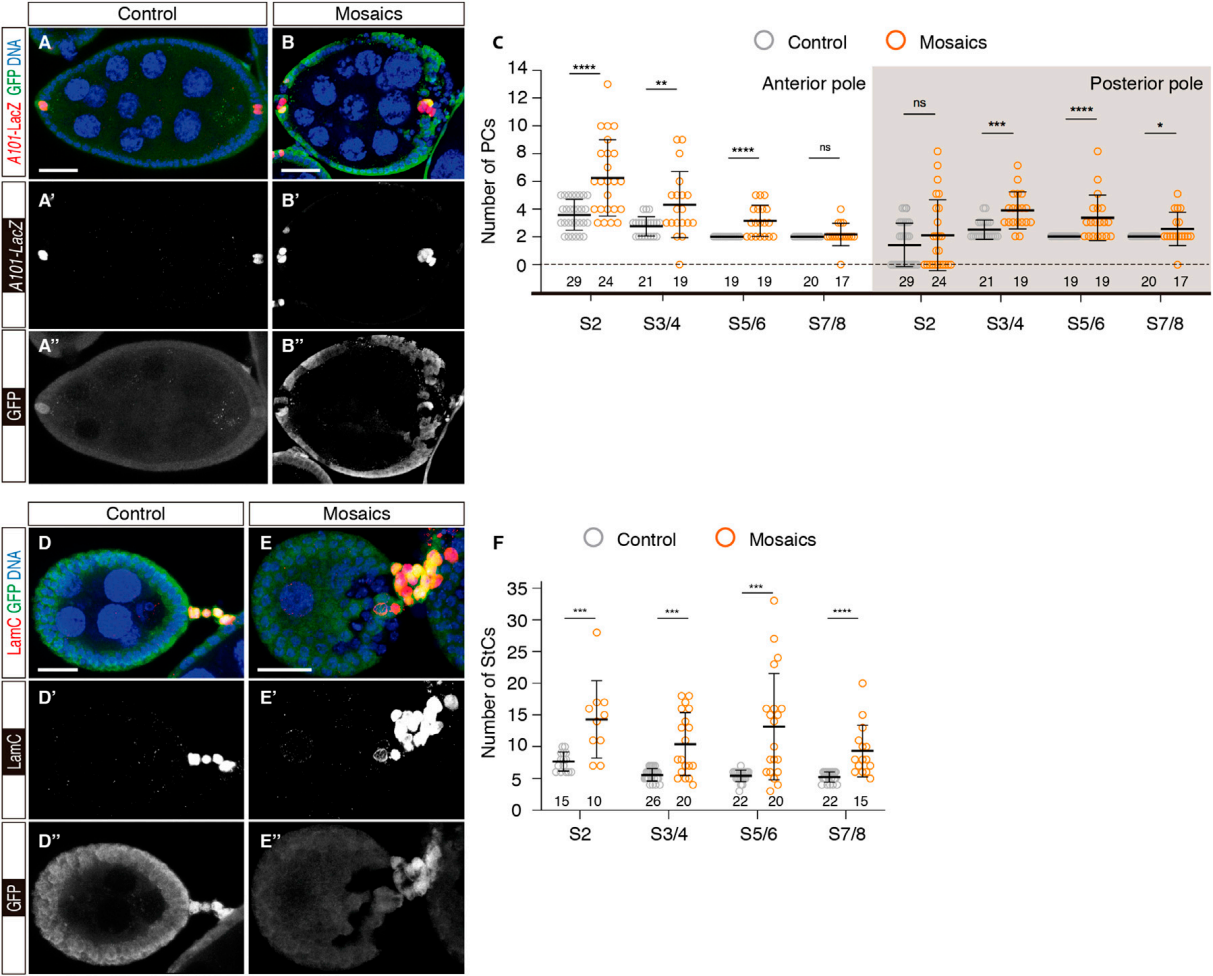
Mosaic epithelia comprising *mys* ^*−*^ cells contain excess polar and stalk cells. **(A–A″)** S7/8 control egg chamber and **(B–B″)** S7/8 experimental egg chamber containing *mys* mutant cells stained to show DNA (blue), GFP (green) and PCs (red). **(C)** Quantification of the number of PCs at the anterior and posterior poles of control and mosaic egg chambers. **(D–D″)** S5/6 control egg chamber and **(E–E″)** S5/6 experimental egg chamber containing *mys* mutant cells stained to show DNA (blue), GFP (green) and StCs (red). Note the abnormal cellular arrangement of the mosaic stalk in **(E)**. **(F)** Quantification of the number of StCs in control and mosaic stalks. Mutant cells are identified by their lack of GFP expression. Digits below the plots correspond to the number of egg chambers analysed (n). ns: not significant. *p* values of unpaired Mann-Whitney U tests considered statistically significant between different genotypes are shown (*: *p* ≤ 0.05, **: *p* ≤ 0.005, ***: *p* ≤ 0.0005, ****: *p* ≤ 0.0001). Scale bars: 20 µm.

Control epithelia containing no *mys* ^*-*^ cells showed the stereotypical drop in PC numbers, with aPCs gradually reducing their numbers from an average of 3.59 ± 0.21 (number of egg chambers analysed (n) = 29) at S2 to only 2 from S5/6 onwards (*n* = 19). S3/4 pPC clusters displayed on average 2.48 ± 0.15 cells (*n* = 21), while it was restricted to 2 cells as from S5/6 (*n* = 19). In contrast, mosaic epithelia containing clones of *mys* ^*-*^ cells at the anterior or posterior ends presented significantly larger PCs numbers at different stages. Thus, aPCs in mosaic epithelia amounted to 6.25 ± 0.56 (*n* = 24) at S2 or 3.16 ± 0.26 (*n* = 19) at S5/6. Similarly, mosaic pPC clusters contained on average 3.84 ± 0.3 cells (*n* = 19) at S3/4 or 3.32 ± 0.37 (*n* = 19) at S5/6. However, the average number of both anterior and posterior PCs in mosaic egg chambers decreased closer to 2 cells at S7/8 (aPCs = 2.18 ± 0.2, *n* = 17; pPCs = 2.53 ± 0.28, *n* = 17) (Figures 2A–C). From these data we conclude that loss of integrin activity in follicle cells at the poles of the egg chamber induces extra anterior and posterior PCs. Moreover, since S2 mosaic chambers already contain more PCs than controls, we surmise a role for integrins in the initial establishment of PC precursors during germarial stages.

One of the Jak/Stat ligands, Unpaired, is transcribed in PCs since S2 and in aPCs it induces border cell (BC) fate in a group of 6-8 surrounding FCs (Grammont and Irvine, 2001; Silver and Montell, 2001; Beccari et al., 2002; Behr et al., 2003). Because mosaic epithelia contained more aPCs until S5/6, we wondered whether these extra aPCs could induce larger than normal BC clusters. In fact, when scoring the number of BCs in control and mosaic S9 and S10 egg chambers, we found that controls contained 6-8 cells (*n* = 36), while BC clusters in mosaic egg chambers carrying extra aPCs always comprised >8 BCs (*n* = 42). These results indicate that BC recruitment started prior to S7/8. In addition, mosaic BC clusters were fragmented in several groups, each of them carrying at least one aPC (Supplementary Figures S2A, B, D).

The analyses of StCs from control and mosaic ovarioles indicated that loss of integrin function induced an increase in StC numbers as well as a disorganisation of the interfollicular stalks. As previously reported, the number of StCs in control ovarioles decreased appreciably from S2 (7.67 ± 0.39, *n* = 15) to S3/4 (5.58 ± 0.19, *n* = 26), after which the number of StCs did not change significantly (S5/6 = 5.41 ± 0.19, *n* = 22; S7/8 = 5.23 ± 0.17, *n* = 22). In contrast, mosaic interfollicular stalks composed of control and *mys* ^*-*^ cells contained significantly higher cell numbers at all stages analysed (S2 = 14.3 ± 1.93, *n* = 10; S3/4 = 10.42 ± 1.14, *n* = 20; S5/6 = 13.15 ± 1.87, *n* = 20; S7/8 = 9.33 ± 1.05, *n* = 15) (Figures 2D–F). As in the case of the PCs, the fact that mosaic S2 stalks encompassed almost twice the number of cells compared to controls also points to a role for integrins in the regulation of stalk cell precursors in the germarium. Finally, we observed a high incidence of StC column disorganisation in mosaic samples, indicating that integrin activity was required for proper stalk formation during the budding off of new egg chambers from the germarium [Figure 2E; (Lovegrove et al., 2019)].

Last, because correct follicular epithelium morphogenesis in the germarium requires the Laminin-binding αPS1βPS integrin (Fernández-Miñán et al., 2007; Akiyama et al., 1994; O'Reilly et al., 2008), we analysed the consequences of a reduction in Laminin levels in the BM surrounding the developing egg chambers. Using an RNA interference construct against the Laminin β1 subunit, common to all known *Drosophila* Laminin trimers, we decreased Laminin levels in experimental ovarioles utilising the *traffic jam-Gal 4* line [*tj > LanB1 RNAi*; (Diaz de la Loza et al., 2017)] and scored PC and StC numbers. We found that, while control aPCs (S2 = 4 ± 0.21 cells, *n* = 22; S5/6 = 2, *n* = 19) and pPCs (S3/4 = 2.35 ± 0.13, *n* = 20; S5/6 = 2, *n* = 19) gave expected values, a notable reduction in Laminin levels induced a significant increase in the number of both, aPCs (S2 = 5.82 ± 0.37, *n* = 22; S5/6 = 2.65 ± 0.21, *n* = 20) and pPCs (S3/4 = 3 ± 0.13, *n* = 20; S5/6 = 2.85 ± 0.21, *n* = 20) (Supplementary Figure S3A–C). Similar to the case of mosaic epithelia, *tj > LanB1 RNAi* egg chambers displayed supernumerary BCs, distributed in several migrating clusters that contained at least one aPC (Supplementary Figures S2C, D).

Regarding the organisation of the interfollicular stalks in *tj > LanB1 RNAi* ovarioles, we found that, while the number of StCs in control ovarioles also followed the reported decrease from S2 to S4, after which StC numbers remained constant (S2 = 8.26 ± 0.36, *n* = 19; S3/4 = 5.89 ± 0.42, *n* = 18; S5/6 = 5.44 ± 0.43, *n* = 16; S7/8 = 6.06 ± 0.29, *n* = 17), *tj > LanB1 RNAi* stalks in contrast were composed of significantly higher cell numbers from S2 onwards (S2 = 9.94 ± 0.57, *n* = 16; S3/4 = 8.61 ± 0.57, *n* = 23; S5/6 = 7.61 ± 0.37, *n* = 23; S7/8 = 7.70 ± 0.61, *n* = 20) (Supplementary Figures S3D–F). From the analyses of mosaic epithelia and of Laminin-depleted samples, we conclude that the integrin-BM interaction has an essential function to control the correct determination of PCs and StCs.

### 3.2 Interfollicular stalks and PC clusters in mosaic epithelia exit the cell cycle and undergo apoptosis like controls

**FIGURE 3:**
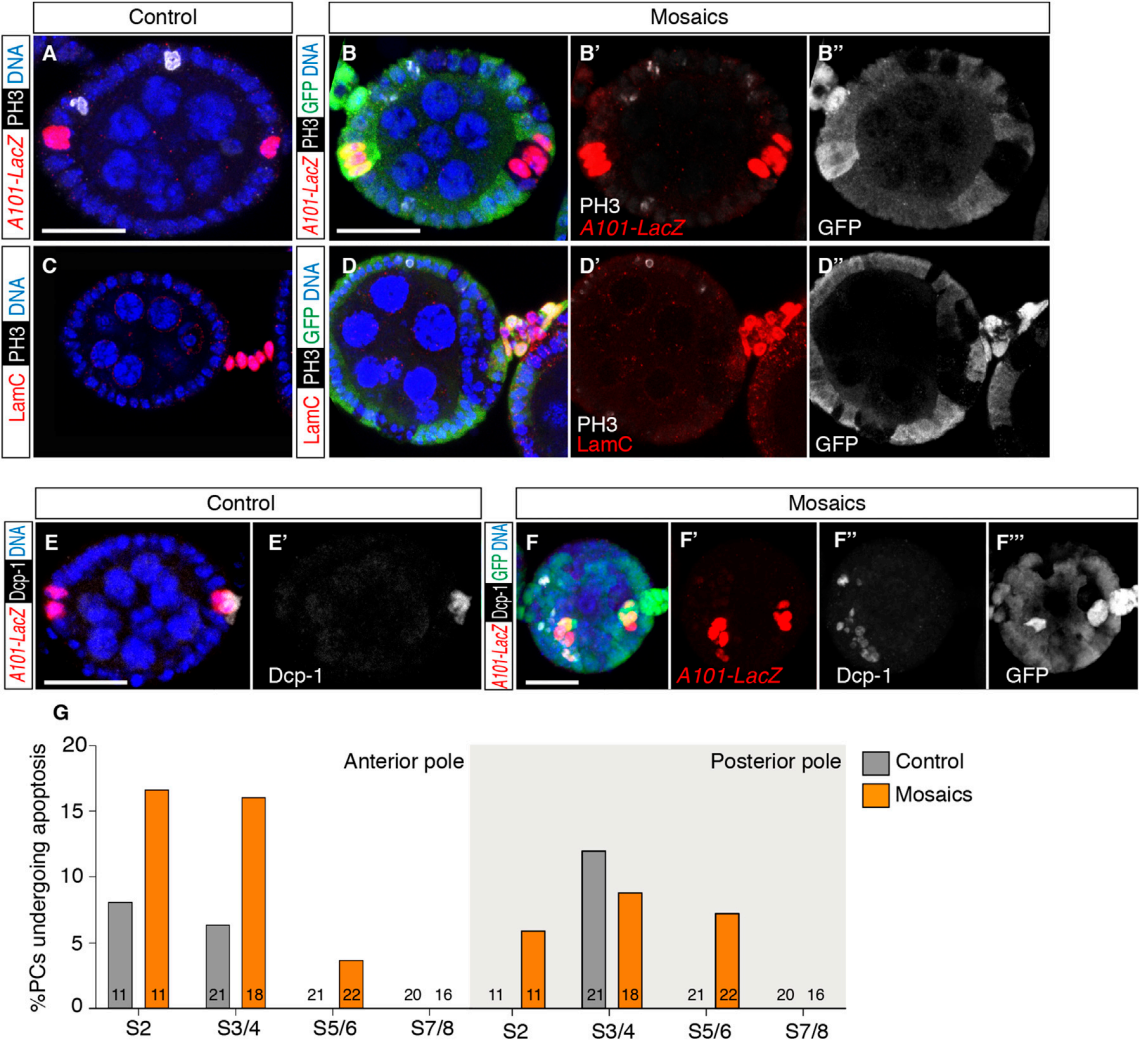
Loss of integrins does not induce PC or StC proliferation after S2 but it causes a higher incidence of cell death. **(A)** S3/4 control egg chamber and **(B–B″)** S3/4 experimental egg chamber containing *mys* ^*+*^ and *mys* ^*−*^ cells stained to show DNA (blue), phospho-Histone H3 (PH3; cells in mitosis; white), GFP (green) and PCs (red). **(C)** S3/4 control egg chamber and **(D–D″)** S5/6 experimental egg chamber containing *mys* ^*+*^ and *mys* ^*−*^ cells stained to show DNA (blue), PH3 (white), GFP (green) and StCs (red). Note the absence of PH3 signal in PCs and StCs of both, control and mosaic samples. Only the control egg chamber in **(A)** contains PH3^+^ cells. **(E,E′)** S5/6 control egg chamber and **(F–F‴)** S3/4 experimental egg chamber containing *mys* ^*+*^ and *mys* ^*−*^ cells stained to show DNA (blue), cell death (Dcp-1; cells in apoptosis; white), GFP (green) and PCs (red). **(G)** Quantification of the number of PCs at the anterior and posterior poles of control and mosaic egg chambers positive for Dcp-1. Digits in the bars correspond to the number of egg chambers analysed (n). Mutant cells are identified by their lack of GFP expression. Scale bars: 20 µm.

Considering that mosaic epithelia contained excess PCs and StCs, we wished to determine if loss of integrin function induced extra PC and StC proliferation after S2, when control cells stop dividing. Using the phospho-Histone H3 antibody as a marker for cells in mitosis, we could observe neither aPCs nor pPCs in mitosis in control (*n* = 77) or in mosaic epithelia (*n* = 68) of S2–S8 egg chambers. Similarly, we could not detect ectopic StC division after S2 in control or experimental samples (*n* = 85 and *n* = 65, respectively) (Figures 3A–D).

Next, knowing that supernumerary PCs undergo programmed cell death to reduce their numbers from the initial ∼4 aPCs present in S2 egg chambers to the final 2 cells found at either pole in S5/6 follicles (Borensztejn et al., 2013), we scored the percentage of apoptotic PCs in control *versus* mosaic epithelia utilising the Dcp1 marker. We found that both anterior and posterior PC clusters of mosaic epithelia analysed at stages 2, 3/4, 5/6 and 7/8 showed consistently higher percentages of Dpc1-positive PCs (Figures 3E–G; controls, *n* = 73 egg chambers analysed; mosaic, *n* = 67). This augmented PC apoptosis in mosaic follicles may explain why, in spite of containing nearly twice as many PCs at S2 compared to controls, the number of PCs in S7/8 experimental egg chambers is not significantly different from controls. We suggest that the specification of extra PCs in mosaic epithelia triggers similar regulatory mechanisms to adjust PC numbers as in normal development—including scarce programmed cell death -, and that these are efficient enough as to achieve the correct complement of PCs by stages 7/8.

We also studied StC apoptosis in both control and mosaic S2-7/8 egg chambers but, due to the very low frequency of Dcp1-positive stalk cells in both, controls (0 Dcp1-positive StCs, *n* = 354 cells analysed from 63 stalks) and mosaic (9 Dcp1-positive StCs, *n* = 744 cells analysed from 65 stalks), we could not draw any statistically significant conclusions from our data.

### 3.3 Increased somatic cell proliferation in mosaic germaria

**FIGURE 4:**
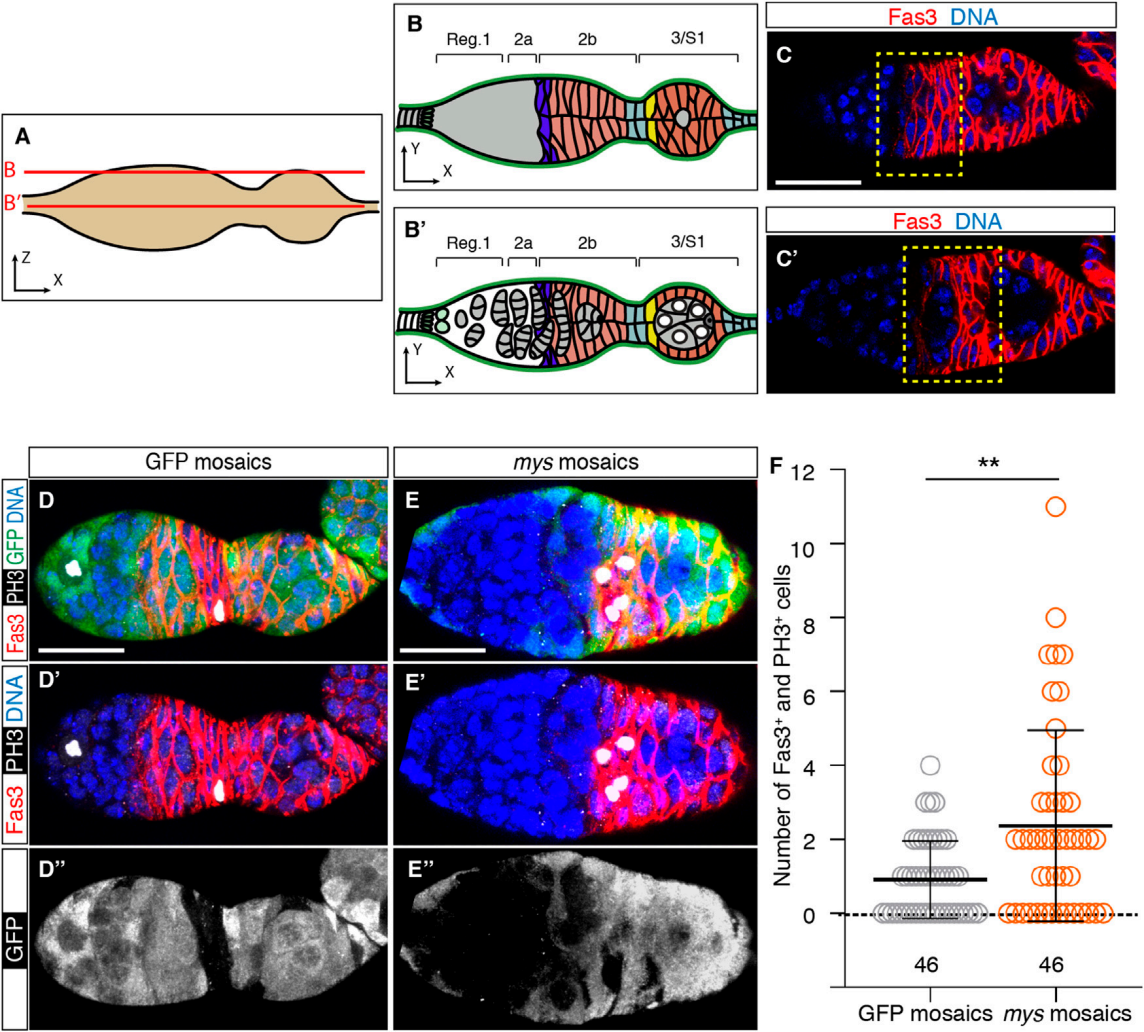
Mosaic germaria show higher incidence of cell proliferation. **(A)** Scheme indicating the confocal sections related to **(B,C′)**. **(B,B′)** Schemes showing the different germarial regions at the surface **(B)** and equatorial **(B′)** planes. **(C,C′)** Confocal images taken at the surface **(C)** and equator **(C′)** of a control germarium showing the pattern of expression of the Fas3 protein, which labels FSCs, PC/StC and FC precursors, and FCs (red). The yellow dotted line indicates regions 2a/2b to early 3, the area where we quantified cells in mitosis. **(D–D″)** Control (GFP-mosaic germarium) and **(E–E″)** *mys*-mosaic germarium stained to show DNA (blue), GFP (green), PH3 (white) and Fas3 (red). **(F)** Quantification of the number of Fas3^+^ phospho-His H3^+^ cells in regions 2a/2b and early 3 in control and mosaic germaria. Mutant cells are identified by their lack of GFP expression. Digits below the plots correspond to the number of egg chambers analysed (n). *p* values of unpaired Mann-Whitney U tests considered statistically significant between different genotypes are shown (**: *p* ≤ 0.005). Scale bars: 20 µm.

Because S2 mosaic epithelia already display supernumerary PCs and StCs, we hypothesised that integrins regulate follicle stem cell (FSC) and PC and StC precursor proliferation in the germarium (Besse and Pret, 2003). To test this, we analysed somatic cell proliferation in the germarial regions where FSCs and PC/StC precursors are located, from region 2a/2b to early region 3 (Figures 4A–C). We detected mitotic cells with phospho-Histone H3 expression and labelled somatic cells (including FSCs and PC/StC precursors) with an anti-Fas3 antibody (O'Brien et al., 2001). We discovered that the frequency of phospho-His H3^+^ cells was higher in mosaic germaria relative to controls, as 67.40% of experimental samples (31/46) contained Fas3^+^ phospho-His H3^+^, compared to 54.35% of control samples (25/46). Furthermore, we found that the number of double-positive cells per germarium was also significantly increased in mosaic germaria (controls = 0.91 ± 0.16 double-positive cells on average, *n* = 46; mosaic = 2.37 ± 0.38, *n* = 46; Figures 4D–F). From these results we conclude that the presence of *mys* mutant cells increased cell proliferation midway through the germarium, in the region where FSCs and pre-follicle cells reside. In this context, since germaria with lower Laminin levels (*tj > LanB1 RNAi*) showed a significant increase in cell proliferation in regions 2a/b and early 3 (control germaria = 0.79 ± 0.14 double-positive cells on average, *n* = 29; mosaic = 2.49 ± 0.43, *n* = 37; Supplementary Figures S4A–C), we conclude that integrin-mediated cell-BM interaction controls FSC and/or PC and StC precursor proliferation in the germarium.

### 3.4 Ectopic *Notch* pathway activation in mosaic germaria

**FIGURE 5:**
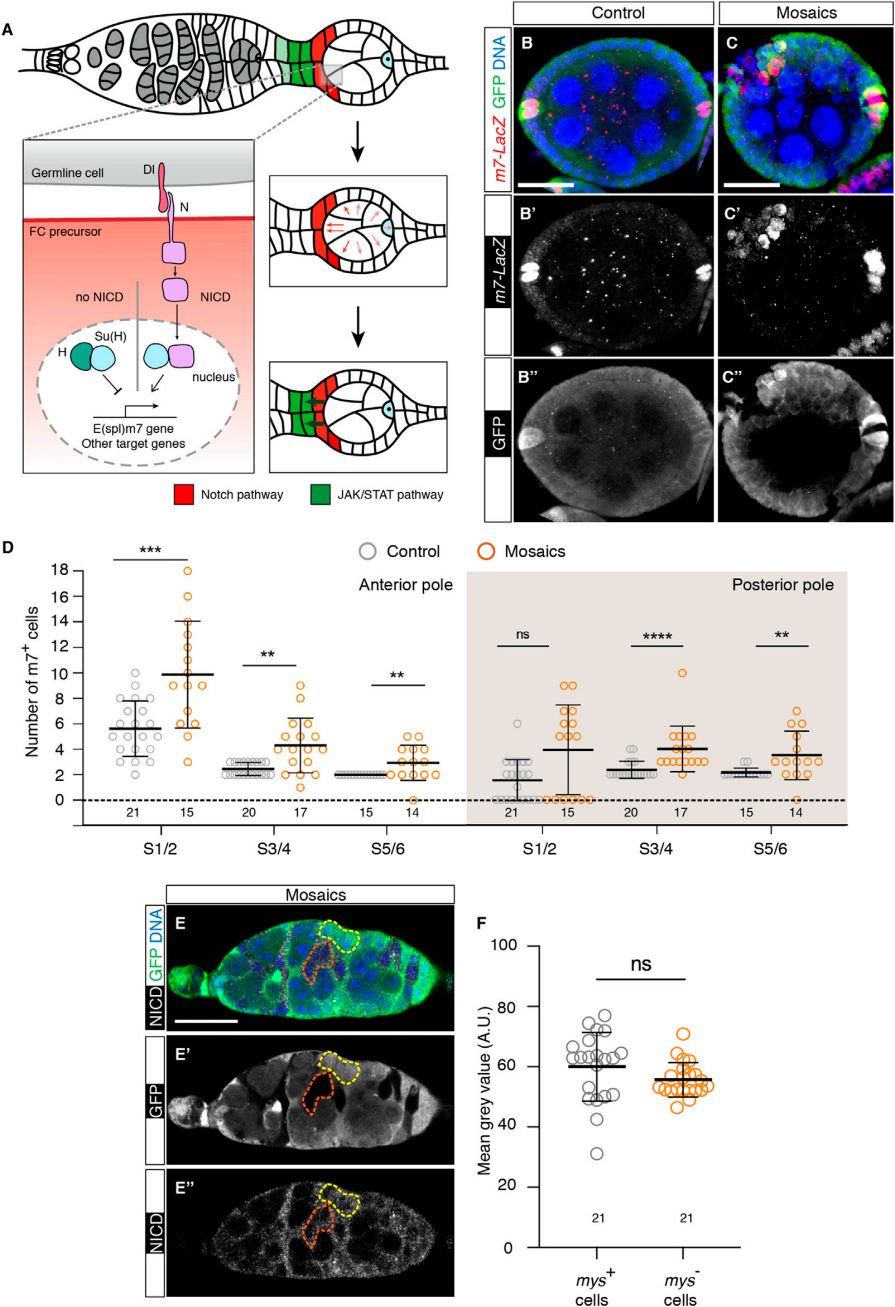
Loss of integrin function in somatic cells in the germarium results in ectopic activation of the Notch pathway. **(A)** Scheme showing the activation of the Notch receptor in anterior S1 somatic cells after receiving the Delta (Dl) signal from germline cells (adapted from (Torres et al., 2003) Processing of the Notch receptor in receiving cells triggers Notch intercellular domain (NICD) release and its nuclear translocation, where it regulates transcription of downstream genes such as *E*(*spl*)*m7*. Notch-transducing cells become PC (red cells). Notch activation in the anterior PCs stimulates JAK/Stat-mediated signalling to adjacent, anterior cells to induce StC fate (green cells). **(B–B″)** Control and **(C–C″)** mosaic S5/6 egg chambers stained to show DNA (blue), GFP (green) and *m7-LacZ* signal (red). **(D)** Quantification of the number of *m7* ^*+*^ cells at the anterior and posterior poles of control and mosaic egg chambers. **(E–E″)** Mosaic germarium stained to show DNA (blue), GFP (green) and NICD (white). The yellow dotted line highlights control cells; the orange dotted line demarcates mutant cells. **(F)** Quantification of the mean grey values (Arbitrary Units) of the selected regions of interest. Digits below the plots correspond to the number of egg chambers **(D)** or ROIs **(F)** analysed (n). ns: not significant. *p* values of unpaired Mann-Whitney U tests considered statistically significant between different genotypes are shown (**: *p* ≤ 0.005, ***: *p* ≤ 0.0005, ****: *p* ≤ 0.0001). Scale bars: 20 µm.

The *Notch* pathway has been implicated in proper cell fate determination in the germarium, including the specification of PC and StC precursors (Figure 5A). In addition, ectopic *Notch* activity results in extra PCs (Grammont and Irvine, 2001; McGregor et al., 2002; Torres et al., 2003; Assa-Kunik et al., 2007; Shyu et al., 2009; Vachias et al., 2010). Considering that mosaic egg chambers contained supernumerary PCs and StCs, we checked whether loss of integrin activity induced ectopic *Notch* signalling. We first utilised the *m7-lacZ* reporter, expressed at high levels in PCs (Assa-Kunik et al., 2007), as a read-out of *Notch* pathway activation and scored the number of high *m7*-expressing cells from S1 to S6 egg chambers. We found that mosaic epithelia consistently showed significantly higher numbers of *m7* ^*+*^ cells at all stages analysed, both at the anterior and posterior poles (n > 14; Figures 5B–D). Second, we quantified the strength of *Notch* signalling by measuring levels of intracellular Notch in *mys* ^*-*^ and control cells in the germarium (Bray, 2006). However, in contrast to *m7* expression, we could not observe significant differences between controls (60 ± 2.49 AU (fluorescence arbitrary units), *n* = 21) and *mys* ^*-*^ cells (55.71 ± 1.24 AU, *n* = 21; Figures 5E, F), suggesting that integrins regulated *Notch* activity independently of Notch intracellular domain levels.

**FIGURE 6:**
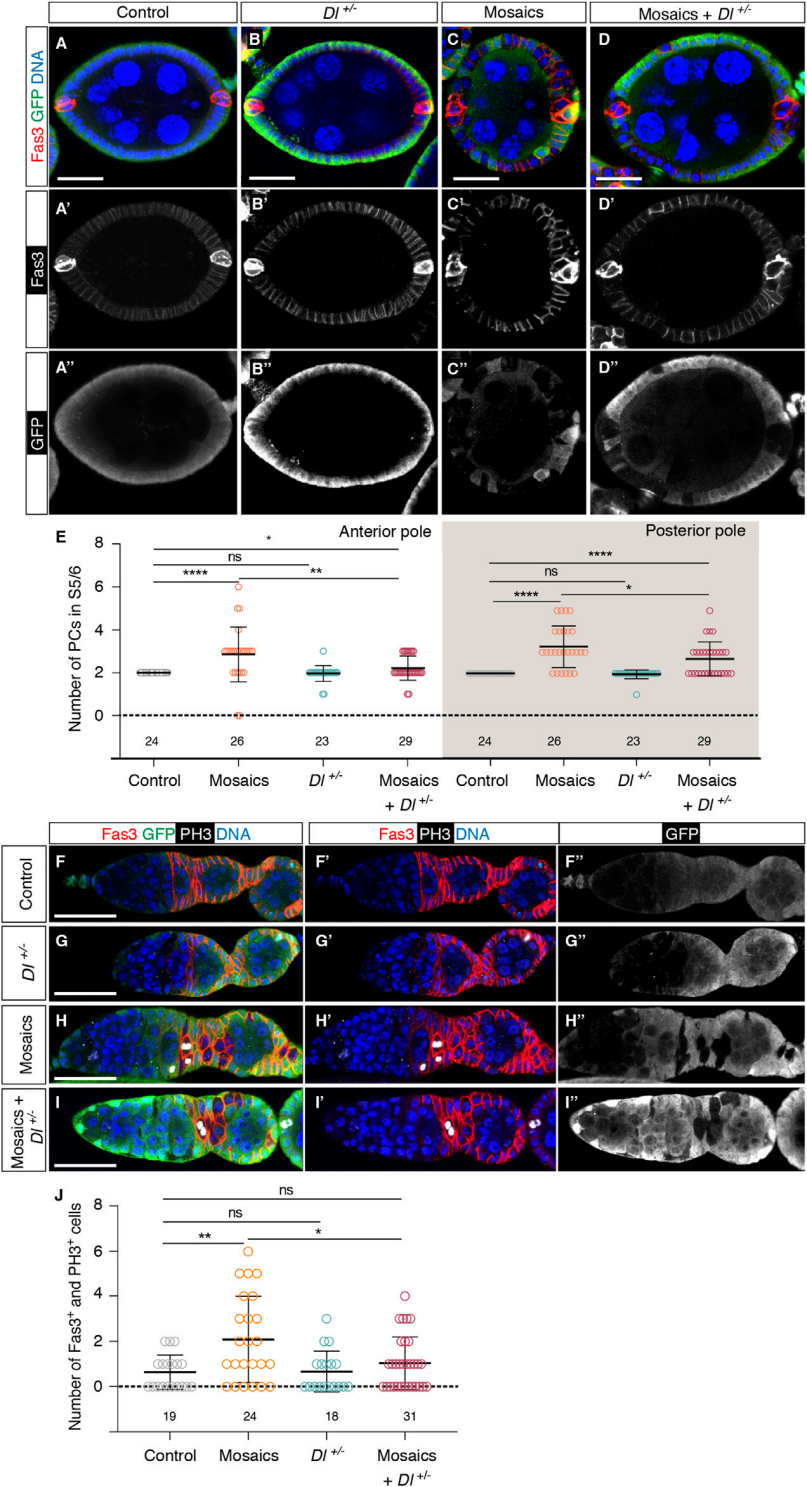
Integrins control Notch pathway activation in the germarium. **(A–A″)** Control, **(B–B″)** *Dl* ^*−*^/+, **(C–C″)** mosaic and **(D–D″)** mosaic in a *Dl* ^*−*^/+ background egg chambers stained to show DNA (blue), GFP (green) and Fas3 (in this occasion, to label PCs; red). **(E)** Quantification of the number of PCs at the anterior and posterior poles of control and experimental egg chambers. **(F–F″)** Control, **(G–G″)** *Dl* ^*−*^/+, **(H–H″)** mosaic and **(I–I″)** mosaic in a *Dl* ^*−*^/+ background germaria stained to show DNA (blue), GFP (green), PH3 (white) and Fas3 (red). **(J)** Quantification of the number of Fas3^+^ phospho-His H3^+^ cells in regions 2a/2b and early 3 in control and experimental germaria. Mutant cells are identified by their lack of GFP expression. Digits below the plots correspond to the number of egg chambers **(E)** or germaria **(J)** analysed (n). ns: not significant. *p* values of unpaired Mann-Whitney U tests considered statistically significant between different genotypes are shown (*: *p* ≤ 0.05, **: *p* ≤ 0.005, ****: *p* ≤ 0.0001). Scale bars: 20 µm.

The above results strongly suggested that integrin function regulated negatively *Notch* pathway activity during early oogenesis. This conclusion was further reinforced by the fact that removing one copy of the *Delta (Dl)* gene, a known ligand of the Notch receptor in the ovary, ameliorated some of the *mys* ^*-*^-associated phenotypes in mosaic epithelia and germaria. Thus, reducing by half the dose of *Dl* in S5/6 mosaic epithelia (mosaic + *Dl* ^*+/−*^) rescued partially, but significantly, aPC and pPC numbers (aPCs in S5/6 mosaic epithelia = 2.85 ± 0.25; mosaic + *Dl* ^*+/−*^ = 2.21 ± 0.10, n > 23; pPCs in mosaic epithelia = 3.27 ± 0.20; mosaic + *Dl* ^*+/−*^ = 2.69 ± 0.15, n > 23) (Figures 6A–E). Moreover, halving *Dl* levels also rescued the germarial proliferation phenotype (control germaria = 0.63 ± 0.18 Fas3^+^ phospho-His H3^+^ cells; mosaic germaria = 2.08 ± 0.38; mosaic + *Dl* ^*+/−*^ = 1.03 ± 0.21, n > 18; Figures 6F–J). From the above results we conclude that integrin-mediated somatic cell-BM interaction(s) regulates *Notch* pathway activation at the poles of S1-S6 egg chambers.

## 4 Discussion

The spatial and temporal coordination between cell cycle withdrawal and differentiation of stem cells is crucial for normal growth and development, and remains decisive for tissue homeostasis and regeneration. The regulation of SC behavior involves the integration of intrinsic and extrinsic cues. A key external cue is integrin-mediated adhesion to the ECM. However, while work in a variety of systems has identified a clear role for integrins in defining and shaping SC niches, integrin function(s) in regulating the SC proliferation-to-differentiation switch is more complex and a generalized role in this context is unlikely to exist. In this study, we show that the elimination of integrins from FSCs and their undifferentiated progeny results in increased proliferation. This is accompanied by an excess of various differentiated follicle cell types, including polar, stalk and border cells, suggesting that cell fate determination can occur in the absence of integrins. These phenotypes are similar to those found in ovarioles with decreased laminin levels, pointing to a role for the integrin-mediated cell-laminin interactions in the control of epithelial division and fate acquisition. In addition, we show that integrins regulate FSC and pre-follicle cell proliferation by restraining the activity of the Notch/Delta pathway.

The role of β integrins in regulating SC proliferation seems to be cell-type specific and somehow unpredictable, highlighting the need to study it in a cellular context-dependent manner [reviewed in (Prowse et al., 2011) and (Watt, 2002)]. Thus, while conditional deletion of β1 integrins in the intestinal epithelial cells caused increased epithelial SC proliferation, its elimination in epidermal and mammary epithelia caused reduced epithelial SC proliferation (Brakebusch et al., 2000; Raghavan et al., 2000; Faraldo et al., 2001). A major difference between the deletion of β1 integrin in the intestinal epithelium and that in epidermal or mammary epithelium is that the structure of the basement membrane was disrupted in the epidermal and mammary epithelia (Brakebusch et al., 2000; Raghavan et al., 2000; Faraldo et al., 2001) but maintained in the intestinal epithelium. Similarly, previous results from our and other laboratories have shown that laminin levels in the *Drosophila* ovary are maintained in early stages with reduced integrin levels (Diaz de la Loza et al., 2017; O'Reilly et al., 2008). Another key factor influencing the behavior of SCs is the composition of the ECM. In various cell types, interactions with fibronectin promotes proliferation and inhibits differentiation, while adhesion to laminin promotes cell cycle withdrawal and morphological and functional differentiation. For example, endothelial cells plated on fibronectin proliferate, but on a laminin-rich matrix they cease growing and rapidly form capillary-like structures (Clark et al., 1982; Kubota et al., 1988). Similarly, myoblasts proliferate on fibronectin, but fuse to form myotubes on laminin (Kuhl et al., 1986). Laminins are also sufficient to stimulate osteogenic differentiation in human mesenchymal SCs (hMSC) (Klees et al., 2005) and differentiation of human induced pluripotent SCs into distinct ocular lineages (Klees et al., 2005). In addition, the onset of adipogenesis is usually defined by ECM remodeling, characterized by the conversion from the fibronectin-rich stromal matrix to the laminin-rich basement membrane and an expression switch from α5 to α6, respective receptors for fibronectin and laminin, at the growth arrest stage of differentiation (Smas and Sul, 1995; Liu et al., 2005). However, while no change in BM composition or integrin expression have been detected during *Drosophila* oogenesis, the expression of the two βPS integrins found in ovaries—PS1 and PS2—increases half way through the germarium, precisely where FSCs reside (O´Reilly et al., 2008). We thus propose that, in the context of FSCs and pre-FCs, the onset of integrin expression and their interaction with laminins triggers exit from proliferation. Our study contradicts two different previously published results. On one hand, an analysis using an RNAi against *mys* showed that downregulation of integrin levels in all somatic cells in the germarium did not alter the number of pre-FCs. The discrepancy between this result and ours may arise from a possible incomplete depletion of *mys* levels when expressing a *mys* RNAi and/or to the lack of competition between control and mutant FSCs, as *mys* is knocked-down in all cells (Lovegrove et al., 2019). Another study reported that removal of integrins or of laminin A—the PS1 ligand—in the adult ovary by heat shock-mediated mitotic recombination in groups of FSCs and pre-follicle cells resulted in their loss after 6 days of clone induction. This cell loss was proposed to be due to reduced FSCs proliferation rate (O´Reilly et al., 2008). The discrepancy between these results and ours may rely on the timing of clone induction and therefore clone size. In our experimental set-up, we started inducing clones in FSCs and some of their progeny at pupal stages and continued throughout adulthood, which resulted in large groups of cells lacking integrins. In contrast, O´Reilly et al. (2008) generated clones by heat-shock in already eclosed female, leading to smaller clones. Hence, our approach eliminates integrin function earlier in oogenesis and induced larger clusters of mutant cells. Altogether, we propose we have uncovered a new, early function for integrin-laminin interaction in the control of cell proliferation in *Drosophila* FSCs and their undifferentiated progeny, which results in excess of PCs and StCs. Interestingly, we find that, at later stages (S7-8), the number of PCs in *mys* mosaic egg chambers is similar to controls. This could be due to the described progressive loss of integrin mutant cells when confronted with wild type cells and/or to the implementation of the mechanisms operating in normal follicular epithelia that ensure the development of only two PCs at each pole. Finally, in agreement with previous results (Bolivar et al., 2006; Lovegrove et al., 2019), we observed a high incidence of StC column disorganisation in mosaic egg chambers. However, while this was interpreted as a requirement of integrins for cellular rearrangement, migration and shape (Bolivar et al., 2006; Lovegrove et al., 2019), here we propose that this phenotype could also arise from excess StC precursor proliferation in absence of integrins. Similarly, mosaic germaria often display aberrant organisation of maturing cysts and the surrounding epithelial cells. Interestingly, since the misshaped stalks, the abnormal multi-layered epithelia and the aberrant germaria typical of mosaic tissues also contain *mys* ^*+*^ cells, it is likely that the presence of *mys* mutant cells affects the behaviour of their *mys* ^*+*^ siblings.

Integrin-mediated adhesion and signaling regulate SC proliferation through cross talk with other signaling pathways [reviewed in (Ellis and Tanentzapf, 2010)]. A major pathway controlling cell proliferation and differentiation in FCs is the Notch pathway. Furthermore, differential strength of pathway activity regulates different aspects of FE proliferation and fate acquisition (Larkin et al., 1996). Thus, while strong ectopic activation of the pathway arrests FCs at a precursor stage, mild or intermediate activation leads to increased number of precursors that further differentiate to polar and stalk cells or in some cases to only stalk cells (Larkin et al., 1996). The latter situation resembles the loss-of-integrin-function phenotype described here. Since the generation of *mys* mutant mosaics induces an overactivation of the Notch pathway and decreasing Notch pathway activity rescues the proliferation and cell fate specification phenotypes caused by the lack of integrins, we thus propose that elimination of integrin function leads to a mild-to-intermediate increase in Notch activation in the germarium. As a consequence, FSCs and pre-FCs over-proliferate and mature into excess polar and stalk cells. We do not know how integrins can restrain Notch pathway activity in FSCs and their progeny, but it differs from what we found in mature posterior follicle cells (Gomez-Lamarca et al., 2014). In this latter case, elimination of integrin function led to defective Notch signaling and endocytosis, thus implicating integrins in Notch intracellular trafficking and/or processing (Gomez-Lamarca et al., 2014). In contrast, here, we find that integrin loss in germarial cells does not alter Notch levels or intracellular localization of NICD. We suggest that in FSCs integrins regulate the Notch pathway downstream of the Notch receptor. Although we do not know how integrins limit Notch activity in FSC and pre-follicle cells, this link between integrins and Notch has been previously described in cultured cells (Deford et al., 2016; LaFoya et al., 2018). In 293T cells, the regulation of Notch by integrins and the ECM is carried out by Src family kinases (SFKs) working downstream of integrins. c-Src directly phosphorylates the NICD at specific tyrosine residues, which attenuates Notch mediated transcription by decreasing recruitment of Mastermind-Like to the Notch co-transcriptional complex (LaFoya et al., 2018). In HMEC cells, blocking antibodies against β1 integrins and β3, which bind laminin and collagen IV, and fibronectin and vitronectin, respectively, enhance Notch dependent transcription. Furthermore, in this context only β3, and not β1, affects accumulation of NICD (Deford et al., 2016). How β1 and β3 integrins differentially control Notch signaling remains unknown. These results together with our observations add to the emerging idea that the cellular microenvironment can determine the mechanism by which integrins couple to Notch signaling.

In summary, we have shown here that a crosstalk between integrin-laminin interactions and the Notch pathway controls the proliferation of a population of FSCs present in the *Drosophila* ovary and their undifferentiated progeny. The use of SCs for clinical applications to treat diseases has stirred an enormous interest in the development of defined microenvironments for SC maintenance, proliferation and subsequent differentiation, to be able to replicate the complex cellular environment existing *in vivo*. Studies over the last years have demonstrated this to be a complicated and challenging goal, as a large variety of SC responses are elicited depending on the cell type and state, repertoire of ECM components and integrins being expressed and their crosstalk with other signaling pathways. However, besides the complexity, some patterns of SC behavior associated to specific cellular contexts seem to be emerging. Thus, interactions with laminins in many cell types, both from vertebrate [reviewed in (Prowse et al., 2011)] and *Drosophila* (this study), seem to very often restrain cell proliferation. Increasing our analysis of SC behavior in different cellular contexts might allow us in the future to associate sets of cellular and environmental factors to specific SC responses, which will be decisive when trying to derive SCs to specific differentiated populations for their application in the clinic.

## Data Availability

The original contributions presented in the study are included in the article/Supplementary Material, further inquiries can be directed to the corresponding authors.

## Supplementary material

SUPPLEMENTARY FIGURE S1 The *e22c-Gal4* line induces mitotic clones during pre-adult stages. **(A,A′)** Mosaic germarium and egg chamber containing *mys* mutant cells from a freshly-eclosed female (0–12 h after eclosion) stained to show DNA (blue) and GFP (green). Mutant cells are identified by their lack of GFP expression. Scale bar: 20 µm.

SUPPLEMENTARY FIGURE S2 Mosaic and Laminin-depleted egg chambers contain supernumerary border cells. **(A,A′)** Control S9 egg chamber, **(B,B′)** mosaic S9 egg chamber and **(C,C′)** Laminin-depleted S10a egg chamber stained to show DNA (blue), GFP (green) and Fas3 (in this occasion, to label PCs in the border cell clusters; red). **(D)** Quantification of the percentage of egg chambers showing normal (6–8) or higher (>8) border cell (BC) numbers. Digits in the bars represent the sample size (n) for each of the genotypes. Yellow arrows point to BC clusters. In all of the panels, they are associated to Fas3^+^ PCs. Scale bars: 20 µm.

SUPPLEMENTARY FIGURE S3 Depletion of Laminin induces excess polar and stalk cells. **(A)** S7/8 control egg chamber and **(B)** Laminin-depleted S7/8 egg chamber stained to show DNA (blue) and PCs (*A101* ^*+*^; red). **(C)** Quantification of the number of PCs at the anterior and posterior poles of control and experimental egg chambers. **(D)** S5/6 control egg chamber and **(E)** S5/6 Laminin-depleted egg chamber stained to show DNA (blue) and StCs (red). Note the abnormal cellular arrangement of the Laminin-knocked down stalk in **(E)**. **(A,B,D)** correspond to single sections; **(E)** is a ∼4-micron projection of several z-planes. **(F)** Quantification of the number of StCs in control and experimental stalks. Digits below the plots correspond to the number of egg chambers analysed (n). ns: not significant. P values of unpaired Mann-Whitney U tests considered statistically significant between different genotypes are shown (*: *p* ≤ 0.05, **: *p* ≤ 0.005, ***: *p* ≤ 0.0005, ****: *p* ≤ 0.0001). Scale bars: 20 µm.

SUPPLEMENTARY FIGURE S4 Mosaic and Laminin-depleted germaria show higher incidence of cell proliferation. **(A)** Control and **(B)** Laminin-depleted germaria stained to show DNA (blue), PH3 (white) and Fas3 (red). **(C)** Quantification of the number of Fas3^+^ phospho-His H3^+^ cells in regions 2a/2b and early 3 in control and Laminin-depleted germaria. Digits below the plots correspond to the number of germaria analysed (n). P values of unpaired Mann-Whitney U tests considered statistically significant between different genotypes are shown (**: *p* ≤ 0.005). Scale bars: 20 µm.
