# Supplementary figures and images for: Integrins control epithelial stem cell proliferation in the Drosophila ovary by modulating the Notch pathway
Source: Front Cell Dev Biol. 2023 Feb 28;11:1114458. doi: 10.3389/fcell.2023.1114458 (PMC10011466; doi:10.3389/fcell.2023.1114458)

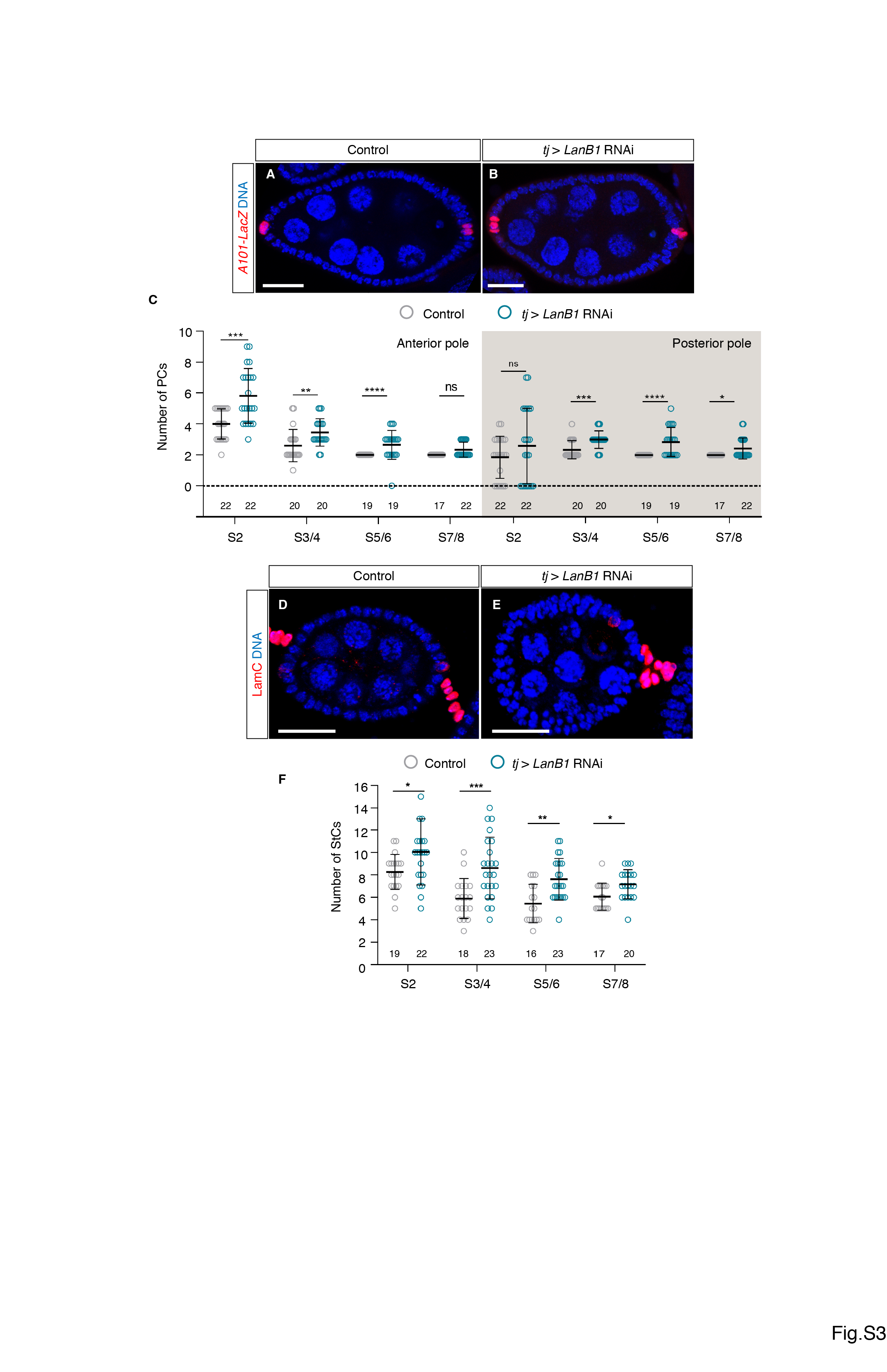

Supplement: Supplementary file 1 [file Image3.tif]

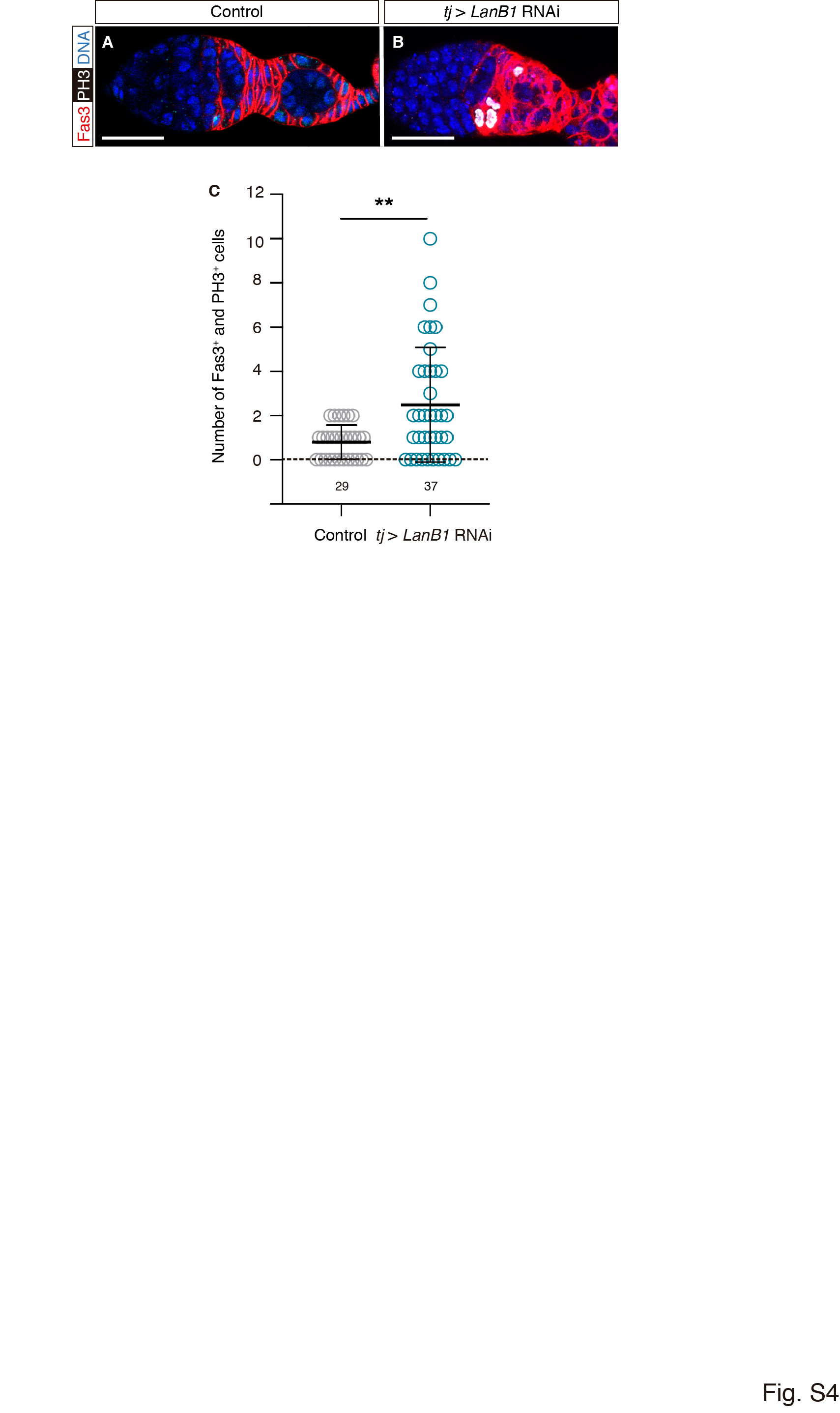

Supplement: Supplementary file 2 [file Image4.tif]

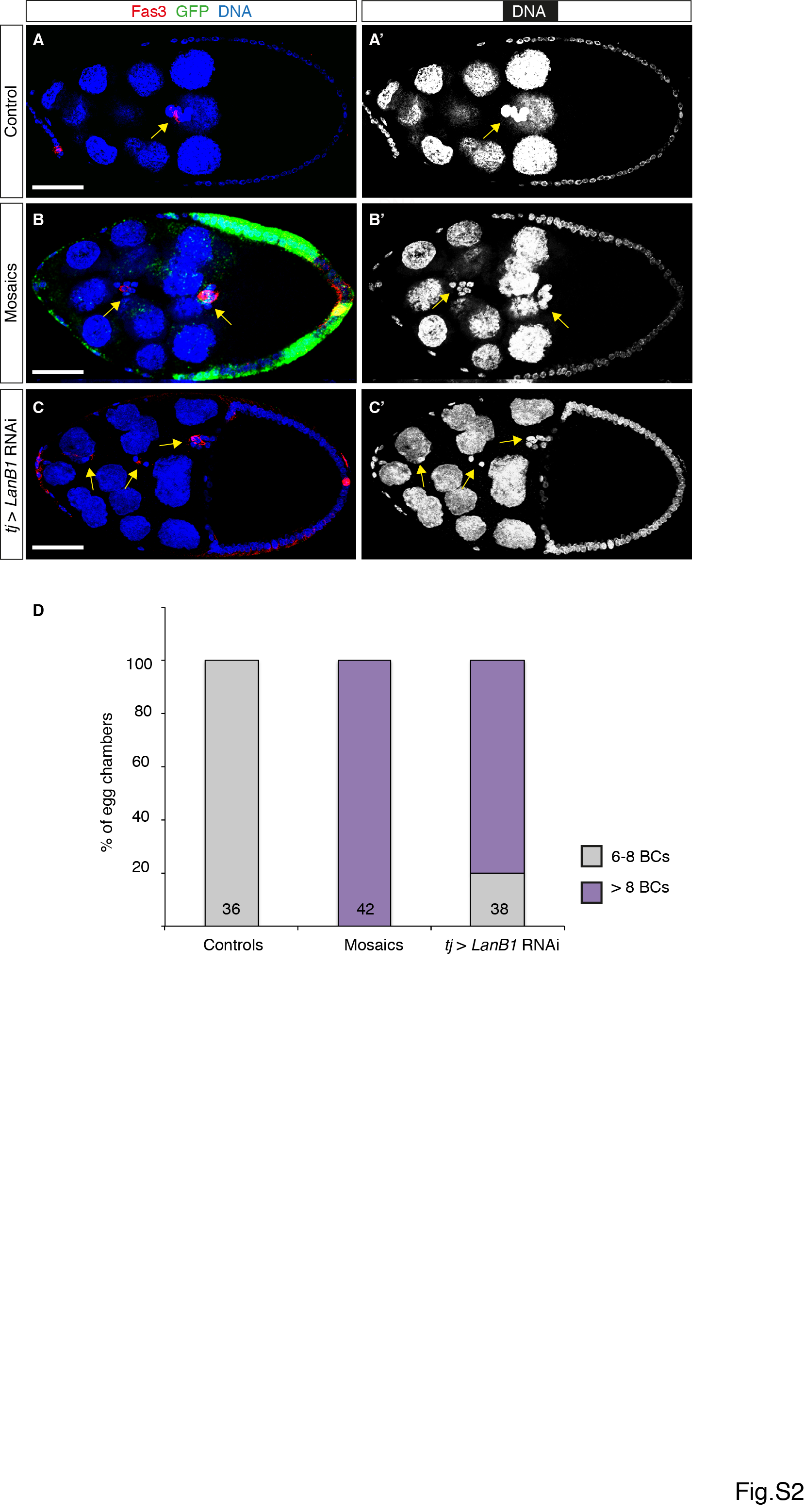

Supplement: Supplementary file 3 [file Image2.tif]

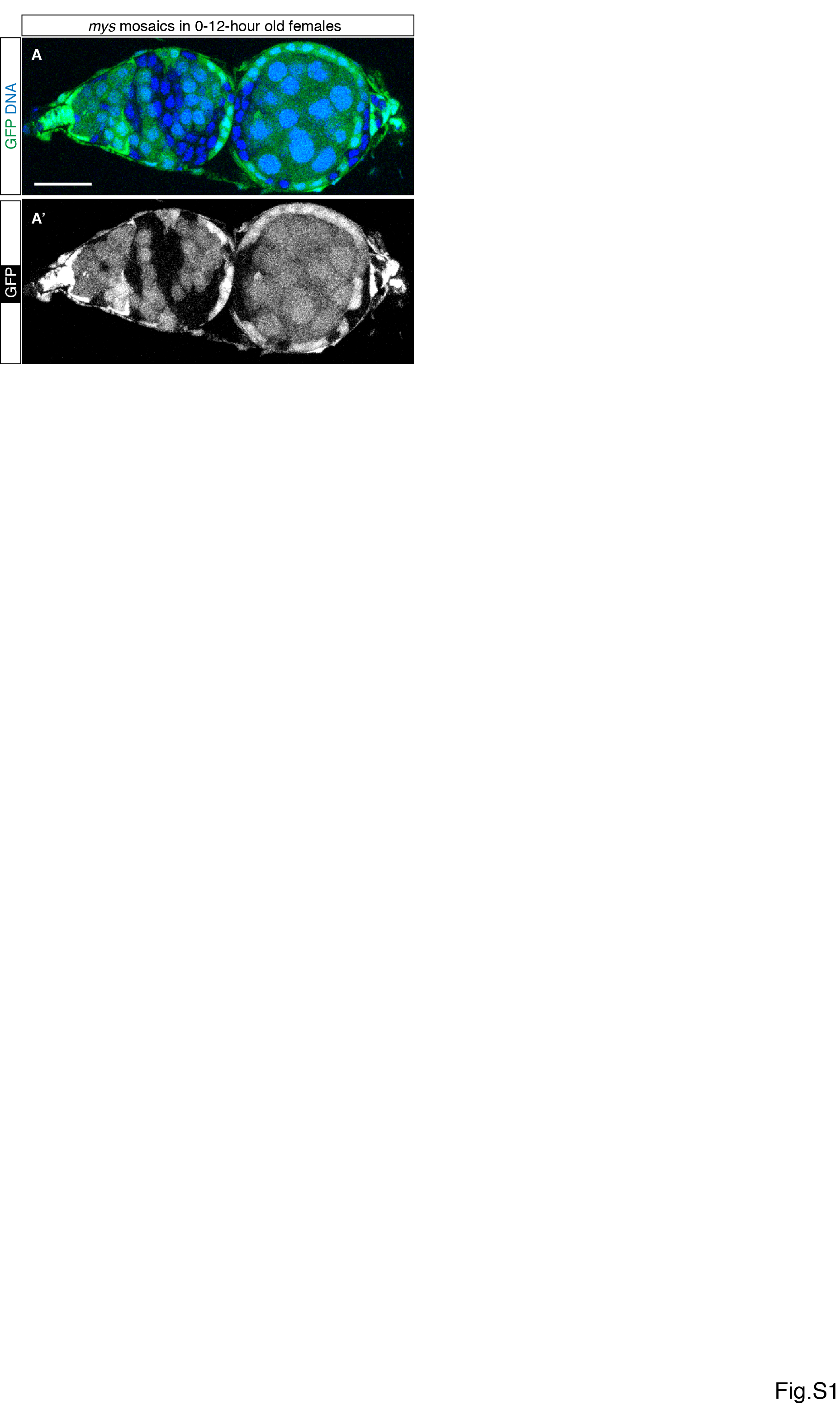

Supplement: Supplementary file 4 [file Image1.tif]
